# Antibacterial Activity of AXOTL-13, a Novel Peptide Identified from the Transcriptome of the Salamander *Ambystoma mexicanum*

**DOI:** 10.3390/pharmaceutics16111445

**Published:** 2024-11-12

**Authors:** Laura Córdoba, Daniela López, Mariana Mejía, Fanny Guzmán, Dina Beltrán, Belfran Carbonell, Laura Medina

**Affiliations:** 1 Grupo Genética, Regeneración y Cáncer, Facultad de Ciencias Exactas y Naturales, Instituto de Biología, Universidad de Antioquia, Medellín 050010, Colombiadalopa99@gmail.com (D.L.); belfran.carbonell@udea.edu.co (B.C.); 2 Núcleo de Biotecnología Curauma (NBC), Pontificia Universidad Católica de Valparaíso, Valparaíso 2373223, Chile; fanny.guzman@pucv.cl (F.G.); dina.beltran.c@mail.pucv.cl (D.B.); 3 Departamento de Estudios Básicos Integrados, Facultad de Odontología, Universidad de Antioquia, Medellín 050010, Colombia

**Keywords:** antimicrobial peptide, antibacterial activity, *Ambystoma mexicanum*, *Escherichia coli*, bioinformatics, axolotl

## Abstract

**Background/Objectives**: Antimicrobial peptides are essential molecules in the innate immunity of various organisms and possess a broad spectrum of antimicrobial, antitumor, and immunomodulatory activities. Due to their multifunctionality, they are seen as an alternative for controlling bacterial infections. Although conventional antibiotics have improved health worldwide, their indiscriminate use has led to the emergence of resistant microorganisms. To discover new molecules with antimicrobial activity that could overcome the limitations of traditional antibiotics, this study aimed to identify antimicrobial peptides in *Ambystoma mexicanum*. **Methods**: In this study, hypothetical proteins encoded in the *Ambystoma mexicanum* transcriptome were predicted. These proteins were aligned with peptides reported in the Antimicrobial Peptide Database (APD3) using the Fasta36 program. After identifying peptide sequences with potential antibacterial activity, their expression was confirmed through conventional polymerase chain reaction (PCR) and then chemically synthesized. The antibacterial activity of the synthesized peptides was evaluated against *Staphylococcus aureus* ATCC 25923 and *Escherichia coli* ATCC 25922. **Results**: A new antimicrobial peptide named AXOTL-13 was identified. AXOTL-13 is an amphipathic cationic alpha-helical peptide with the ability to inhibit the growth of *Escherichia coli* without causing hemolysis in red blood cells, with its action likely directed at the membrane, as suggested by morphological changes observed through scanning electron microscopy. **Conclusions**: This research is pioneering in evaluating the activity of antimicrobial peptides present in *Ambystoma mexicanum* and in specifically identifying one of these peptides. The findings will serve as a reference for future research in this field.

## 1. Introduction

The innate immune system constitutes the first line of defense that protects against infections caused by pathogens found in the environment. This system has a long evolutionary heritage as it consists of defense mechanisms shared across species ) [1,2]. Among the most important compounds and primitive mechanisms conserved and used by both eukaryotic and prokaryotic cells are antimicrobial peptides (AMPs), which are key effector molecules of chemical defense widely distributed in nature, from bacteria and archaea to fungi, plants, and animals [3,4,5].

AMPs are a group of molecules with broad-spectrum antimicrobial properties that play a fundamental role in host protection against bacteria, viruses, fungi, and protozoa. Additionally, these molecules may have antitumor and immunomodulatory properties, leading to the use of the term “host defense peptides” [6,7,8]. In nature, there is a wide variety of peptides, diverse in size, secondary structure, and physicochemical properties [9]. Despite their heterogeneity, many of these molecules are cationic, with a net charge ranging from +2 to +13, and 50% of their constituent amino acids are hydrophobic, making them mostly amphipathic molecules [10]. As a result, AMPs exhibit multiple mechanisms of action that can be divided into two main groups: immune modulation and direct killing mechanisms. Regarding direct killing, peptides can interact with the cell membrane, compromising its integrity and causing cell lysis or interfering with various intracellular functions, including replication, transcription, translation, protein folding, and cell division, among others [10,11].

Their multifunctionality has led to the utilization of AMPs for the benefit of human health, positioning these molecules as a potential strategy for controlling bacterial infections [8]. This is supported by the emergence and spread of drug-resistant pathogens, which continue to compromise our ability to treat common infections, becoming a global health problem [12,13]. The World Health Organization (WHO) has ranked antimicrobial resistance among the top ten global threats to public health. It anticipates that by 2050, it will cause around 10 million deaths annually, underscoring the need to identify new active compounds and suggesting AMPs as promising alternatives, given their limited development of resistance over millions of years [13]. This has spurred research on the isolation and characterization of peptides, leading to a rapid increase in the number of these molecules and the need for methodologies that aid in their registration and classification) [14,15]. In response to this need, databases have emerged to compile published information on peptides. APD3 offers comprehensive data and is a valuable tool for understanding antimicrobial peptides (AMPs). Currently, it contains over 3500 peptides with experimentally verified activity [16].

Most AMPs have been identified in amphibians, with approximately 33% of the peptides registered in the APD3 database originating from these animals [16]. This figure is primarily explained by the biological adaptation that amphibian skin has undergone to environmental changes [17]. As a result, the skin not only acts as a primary immunological barrier to the environment but also contains mucous and granular glands, which are complex structures with versatile functions. The granular glands serve as a reservoir and source of secretion of AMPs for protection against predators or microorganisms [17]. In addition, it has been found that other tissues and organs secrete these molecules. The intestine, for example, contains epithelial cells that express a variety of AMPs, which play a fundamental role in infection control and the maintenance of a functional and stable microbiota [18,19,20]. Furthermore, heart cells have also been implicated in the production of AMPs, showing that these peptides not only possess antibacterial properties [21] but are also involved in cardiac activity [22].

According to the APD3 database, around 1079 peptides have been identified in amphibians, with 1006 in frogs and 68 in toads. However, the number significantly decreases when referring to the order Caudata [16]. This demonstrates the presence of AMPs in this last taxonomic category but only a few studies have been dedicated to their discovery. In the Ambystomatidae family of the Caudata order, only one study of peptides with antimicrobial activity identified in the skin of the tiger salamander (*Ambystoma tigrinum*) has been reported [23]. There are no confirmed reports of antimicrobial peptides (AMPs) in the species *Ambystoma mexicanum*, belonging to the Ambystomatidae family. Although some peptides with potential antimicrobial activity have been suggested, they have not been evaluated or confirmed, highlighting a significant gap in our understanding of the host defense peptides present in this organism [24]. Nevertheless, considering the close evolutionary relationship between *A. tigrinum* and *A. mexicanum*, as well as the conservation of signal peptide and propeptide sequences between families [25,26], the identification of AMPs in the axolotl is quite likely. Furthermore, given that this species retains histological skin structures similar to most amphibians, with mucous and granular glands embedded within the spongy layer, it is to be expected that the axolotl has the same capacity to synthesize and store AMPs, as seen in other amphibians where the majority of these molecules have already been identified [27].

Building upon this, the present research evaluated the antimicrobial effect of new peptides identified in silico from the transcriptome of *Ambystoma mexicanum* (*A. mexicanum*). After conducting in vitro assays, the first antibacterial peptide in this species, AXOTL-13, was discovered. It demonstrated the ability to inhibit the growth of *Escherichia coli* without causing lysis of human red blood cells, positioning it as a potential therapeutic agent.

## 2. Materials and Methods

### 2.1. In Silico Identification of the Potential Antimicrobial Peptides to Be Evaluated

#### 2.1.1. Acquisition of Hypothetical Proteins

The transcriptome derived from amputations of anterior limbs of *A. mexicanum*, published by Dwaraka et al. (2019) [28], is available in the NCBI GEO database with accession number GSE116777. The transcriptome was translated using the TransDecoder v5 4.0 tool (available online: https://github.com/TransDecoder/TransDecoder/wiki (accessed on 18 April 2020)) [29], which identifies coding regions based on the recognition of long amino acid sequences in open reading frames (ORFs), thus achieving the acquisition of hypothetical proteins.

#### 2.1.2. Detection of Candidate Peptides in *A. mexicanum*

A sequence alignment was performed between hypothetical proteins and peptide sequences reported in the APD3 database (available online: https://aps.unmc.edu/, accessed on 1 June 2021) [16] The alignment was performed using a global–local search with Fasta36, a program designed for the comparison of DNA and protein sequences [30]. The multiple sequence alignment, aiming to identify potential AMPs, is based on the percentages of similarity with already reported sequences [31].

#### 2.1.3. Selection of Candidate Peptides

Similar amino acid sequences to those of existing peptides with previously reported effects in the APD3 were selected, considering the eligibility criteria shown in Table 1.

#### 2.1.4. Prediction of the Antimicrobial Activity of the Candidate Peptides

The CAMP R3 web server—Antimicrobial Peptide Collection—was used to predict antimicrobial activity (available online: www.camp3.bicnirrh.res.in/index.php, accessed on 7 October 2021) [32]. This web server has various functions, one of which is the prediction of potential antimicrobial activity. In this regard, the server provides a value between 0 and 1, with a value closer to 1 being indicative of a higher probability that the peptides exhibit antimicrobial activity [32]. In this way, the peptides with potential antimicrobial activity were selected to be chemically synthesized.

#### 2.1.5. Molecular Confirmation of the Candidate Peptides

To confirm the presence of the sequences encoding the candidate peptides in *A. mexicanum*, conventional PCR was performed. This method allowed the amplification of the nucleotide sequences encoding these peptides, using cDNA from the skin, intestine, and heart tissues of the animal. This cDNA was obtained from RNA extraction, according to the procedure previously reported by the Genetics, Regeneration, and Cancer Research Group [33].

#### 2.1.6. Peptide Synthesis

The synthesis of the selected peptides was carried out in the Peptide Synthesis Laboratory at the Curauma Biotechnology Center, using the Fmoc solid-phase chemical synthesis method [34].

### 2.2. In Vitro Assays

#### 2.2.1. Evaluation of the Antimicrobial Activity

To establish the minimum inhibitory concentration (MIC), the microdilution-in-broth protocol for AMPs proposed by Wiegand et al. (2008) [35] was performed. Bacterial strains *Escherichia coli* ATCC 25922 and *Staphylococcus aureus* ATCC 25923 were cultured in tryptic soy agar (TSA). For the assay, 5 bacterial colonies were taken, dissolved in tryptic soy broth (TSB), and incubated at 37 °C until reaching 0.5 on the McFarland scale (1 × 10^8^ CFU/mL). Subsequently, a dilution was made to achieve a final concentration of 5 × 10^5^ CFU/mL. A 96-well plate was used for the assay in which each bacterial strain was exposed to different concentrations of the peptides to be evaluated: 70 μM, 35 μM, 17.5 μM, and 8.7 μM. Different controls were employed: TSB medium served as the sterility control and blank; bacteria without treatment were utilized as the negative control; and bacteria exposed to the Ramosin peptide at 40 μM [31] and sodium hypochlorite at 0.06% were employed as positive controls. Each treatment was performed in triplicate, and each assay was carried out on two different days. The plate was incubated for 20 h at 37 °C in a Multiskan Sky (Thermo Fisher, Waltham, MA, USA), reading the absorbance at a wavelength of 600 nm every hour.

To determine the bactericidal or bacteriostatic effect of the peptides, the procedure dictated by the Clinical and Laboratory Standards Institute (CLSI, 1999) was followed [36]: 5 μL from each well in which no microbial growth was visually observed was taken and streaked onto TSA using the microdot technique. The bacteria were incubated for 24 h at 37 °C, and the absence or presence of bacterial growth was verified.

The results were read in two ways: an endpoint analysis in which the absorbances of the wells were read at 20 h of treatment, to determine the inhibitory effect of the peptide, and a bacterial growth kinetics analysis in which the behavior of the peptide throughout the assay was observed, identifying a bactericidal or bacteriostatic action.

#### 2.2.2. Evaluation of Hemolytic Activity

The determination of the hemolytic activity of the peptide AXOTL-13 was performed as described in previous studies [37]: 3 mL of human blood was collected in BD Vacutainer K2 EDTA tubes, which were centrifuged at 2000× *g* for 10 min at 4 °C. After performing several washes with 1× PBS, 65 μL of the red blood cell suspension was mixed with 65 μL of each peptide concentration (140 μM, 70 μM, 35 μM, 17.5 μM, 8.7 μM, and 4.4 μM) in a 96-well plate. For the negative and positive controls, the erythrocytes were exposed to 1× PBS and Triton X-100 at 0.5% *v*/*v*, respectively. Each treatment was performed in triplicate, and the experiment was conducted on two different days. The plates were incubated for 2 h at 37 °C, then centrifuged at 3000× *g* for 5 min; 80 μL of each supernatant was transferred to a clean well and diluted (1:2) with sterile distilled water. The absorbance was read at 540 nm using a Multiskan Sky, which allowed the measurement of hemoglobin released by the erythrocytes and, thus, the percentage of hemolytic activity, calculated according to the following formula:

hemolysis (%) = (((OD540 sample)/(OD540 positive control Triton X-100)) − 0D540 negative control PBS 1X) × 100

### 2.3. Characterization of the Peptide AXOTL-13

#### 2.3.1. Determination of Physicochemical Properties

To determine the physicochemical properties of AXOTL-13, the NovoPro (2014) peptide property calculator tool was used (available online: https://www.novoprolabs.com/tools/calc_peptide_property, accessed on 9 May 2023) [38], which estimates the chemical formula, extinction coefficient, net charge, isoelectric point, and GRAVY (grand average of hydropathicity index).

#### 2.3.2. Secondary Structure Modeling

Once the physicochemical properties were determined, secondary structure modeling was carried out using the I-TASSER server (available online: https://zhanggroup.org/I-TASSER/, accessed on 9 May 2023) [39,40,41]. In addition, the amino acid arrangement of the peptides was determined through the Schiffer and Edmundson wheel representation by NetWheels (available online: https://github.com/molx/NetWheels, accessed on 9 May 2023) [42].

#### 2.3.3. Circular Dichroism Spectroscopy

To confirm the secondary structure of the peptide AXOTL-13, circular dichroism (CD) analysis was performed using a JASCO J-815 CD spectrometer (JASCO Corp., Tokyo, Japan) in the far-ultraviolet (UV) range (190–250 nm). The molar ellipticity was calculated for each spectrum using 250 μL of AXOTL-13 at a 2 mM concentration in 2% (*v*/*v*) 2,2,2-trifluoroethanol (TFE) and in water. These assays were carried out in the Peptide Synthesis Laboratory at the Curauma Biotechnology Center [43,44].

#### 2.3.4. Scanning Electron Microscopy (SEM)

Aliquots of *E. coli* ATCC 25922 were collected, inoculated in TSB medium, and incubated at 37 °C until reaching the exponential growth phase. The samples were then centrifuged at 2000× *g* for 5 min, removing the supernatant and resuspending the bacterial pellet in PBS. These suspensions were incubated for 15 min at 37 °C in the presence of the peptide AXOTL-13 at a 30 μM concentration. Subsequently, they were centrifuged at 2000× *g* for 5 min; the supernatants were discarded, and glutaraldehyde was added at a 1:3 ratio as a fixative. Untreated bacteria were included as a negative control, and the peptide Ramosin, with previously confirmed activity [31], was used as a positive control. These assays were performed on two occasions and different days. Later, the samples were sent to the scanning electron microscopy service at the University of Antioquia for respective analysis.

### 2.4. Bioethical Considerations

All experimental procedures were approved by the Institutional Committee on Bioethics, Care, and Animal Use of the University of Antioquia (Medellin, Colombia).

### 2.5. Statistical Analysis

To compare the viability percentages obtained after treating the bacteria with different concentrations of each peptide, the assumptions of normal distribution and homoscedasticity of variances were initially verified using the Shapiro–Wilk and Levene statistical tests, respectively, using R software version 4.2.3 [45]. In this way, when the mentioned assumptions were met, a one-way ANOVA test and multiple comparisons using the Tukey test were used. In cases where the assumptions were not met, the non-parametric Kruskal–Wallis test was used for multiple comparisons, and the Mann–Whitney U test was used for comparisons between two groups. These analyses were conducted using GraphPad Prism software version 5.0.0 (2007) [46]. The same statistical tools were also employed to compare hemolytic activity percentages. A *p*-value < 0.05 was considered statistically significant.

## 3. Results

### 3.1. Peptides Identified In Silico in the Transcriptome of Ambystoma mexicanum

Following the application of the inclusion criteria outlined in Table 1, a total of 91 candidate sequences were obtained. These sequences were subjected to antimicrobial effect prediction using the CAMP R3 web server, resulting in the identification of 12 sequences with potential antimicrobial properties. To investigate the expression of these sequences in *A. mexicanum* tissues, conventional PCR was performed using cDNA from the skin, intestine, and heart (Figure S1). Subsequently, those peptide sequences confirmed to be expressed in all three tissues were chemically synthesized by F-moc solid-phase peptide synthesis. The purity of these peptides was confirmed through RP-HPLC analysis (Figure S2), and their molecular weights were validated using electrospray ionization mass spectrometry (ESI-MS) (Figure S3). Detailed characteristics of these peptides are provided in Table 2.

### 3.2. Antimicrobial Activity

During the antimicrobial activity assays, it was observed that peptides 4766, 4767, 4768, 4769, and 4771 did not exert a complete inhibitory effect on the growth of the tested strains. Considering that these peptides did not achieve a significant reduction in the bacterial viability percentage, the results obtained with these peptides are not included in this article. Therefore, further studies were focused on peptide 4770, which was named AXOTL-13, denoting its origin from the axolotl organism and its number of amino acids.

**Figure 1:**
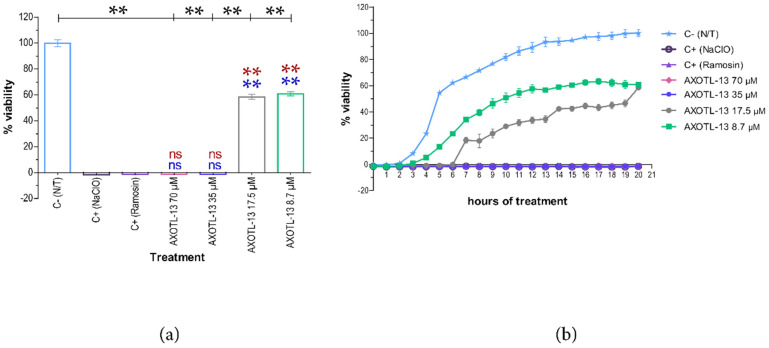
Antibacterial activity of the AXOTL-13 peptide on *Escherichia coli.* (**a**) Viability percentage after 20 h of growth (endpoint). (**b**) Viability percentage every hour for 20 h (growth kinetics). C− (N/T): bacteria without treatment. C+ (NaClO): bacteria treated with 0.06% NaClO. C+ (Ramosin): bacteria treated with 40 μM Ramosin peptide. Kinetics data are presented as mean ± SD and endpoint as mean ± SEM. ns: *p* ≥ 0.005. **: *p* < 0.01. Blue asterisk: comparison with NaClO; red asterisk: comparison with Ramosin.

Regarding peptide AXOTL-13, the endpoint analysis, in which *E. coli* was exposed to this peptide at different concentrations, revealed that it exerted an antibacterial effect on the microorganism. When comparing the effect produced by the peptide at concentrations of 70 μM and 35 μM with the positive controls, there is no statistically significant difference (*p* > 0.05). Additionally, a statistically significant difference is observed when compared to the negative control (*p* < 0.05) (Figure 1a). In contrast, concentrations of 17.5 μM and 8.7 μM showed statistically different results compared to the positive controls (*p* < 0.05) and the negative control (*p* < 0.05). These concentrations did not completely inhibit the growth of the bacterial strain but did reduce its viability by an average of up to 58.5% and 60.8%, respectively. These results suggest the ability of AXOTL-13 to cause bacterial death and, consequently, inhibit its growth at a concentration of 35 μM (minimum inhibitory concentration) (Figure 1a).

Furthermore, the kinetic graph reflects the previously described antibacterial effect. It also shows that peptide AXOTL-13 at concentrations of 70 μM and 35 μM consistently inhibited microbial growth during the 20 h of evaluation, similar to the positive controls (Figure 1b). This indicates a bactericidal action, which was confirmed by inoculating 5 μL of the test wells without bacterial growth in TSA medium and observing the absence of bacterial colonies after 24 h of incubation (Figure S4). As mentioned earlier, no significant difference was observed between 17.5 μM and 8.7 μM in the endpoint analysis. Nevertheless, the kinetic graph shows that during 19 h of treatment, lower concentrations resulted in a higher viability percentage (Figure 1b).

**Figure 2:**
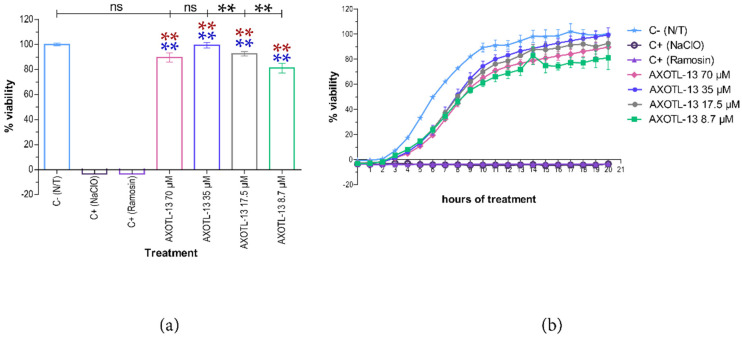
Antibacterial activity of the AXOTL-13 peptide on *Staphylococcus aureus.* (**a**) Viability percentage after 20 h of growth (endpoint). (**b**) Viability percentage every hour for 20 h (growth kinetics). C− (N/T): bacteria without treatment. C+ (NaClO): bacteria treated with 0.06% NaClO. C+ (Ramosin): bacteria treated with 40 μM Ramosin peptide. Kinetics data are presented as mean ± SD and endpoint as mean ± SEM. ns: *p* ≥ 0.005. **: *p* < 0.01. Blue asterisk: comparison with NaClO; red asterisk: comparison with Ramosin.

In contrast to the effect observed in *E. coli*, all evaluated concentrations of peptide AXOTL-13 against *S. aureus* exhibited statistically significant differences with the positive controls (*p* < 0.05). However, when compared to the negative control, concentrations of 70 μM and 35 μM did not show statistically significant differences (*p* > 0.05), while concentrations of 17.5 μM and 8.7 μM did. These results suggest that the peptide does not inhibit the growth of *S. aureus* bacteria at the concentrations and time points assessed (Figure 2a). The kinetic graph shows that over the course of 20 h, all concentrations exhibited a standard bacterial growth curve, including the latent phase, exponential phase, and stationary phase, with no decrease in the number of microbial cells (Figure 2b).

### 3.3. Hemolytic Activity of Peptide AXOTL-13

**Figure 3:**
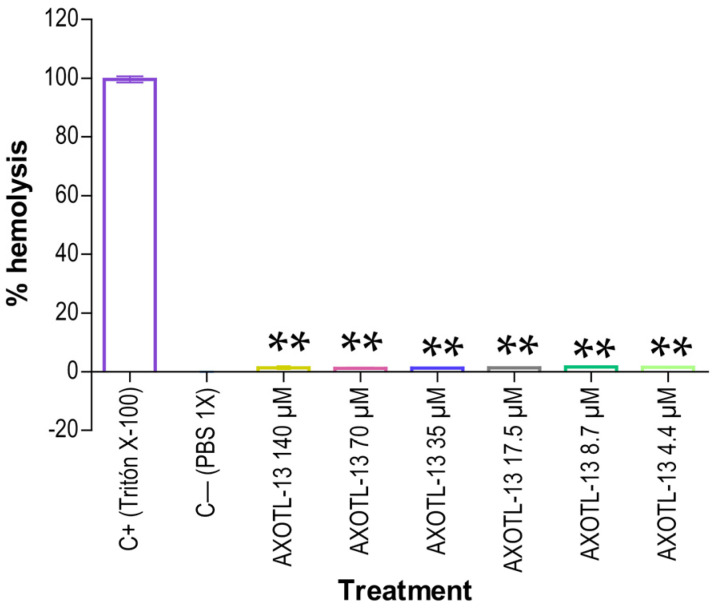
Hemolytic activity of AXOTL-13. C+ (Triton X-100): Red blood cells with 0.5% *v*/*v* Triton X-100. C− (PBS 1X): Red blood cells with PBS 1X. Data are presented as mean ± SEM. **: *p* < 0.01. Black asterisk: comparison with Tritón-X-100.

To assess the hemolytic potential of AXOTL-13 on human red blood cells, the procedure previously reported [37] was followed. As depicted in Figure 3, statistically significant differences (*p* < 0.05) were observed in all evaluated concentrations compared to the positive control. Furthermore, it was noted that the percentage of hemolysis induced by AXOTL-13 ranged from 1.2% to 1.6% across the six examined concentrations. These results suggest that no significant hemolytic effect is observed until a concentration of 140 μM, which exceeds the highest concentration evaluated in the antimicrobial assay.

### 3.4. Physicochemical Properties of Peptide AXOTL-13

Table 3 presents the physicochemical properties of peptide AXOTL-13, as provided by the NovoPro peptide property calculator.

### 3.5. Secondary Structure of Peptide AXOTL-13

**Figure 4:**
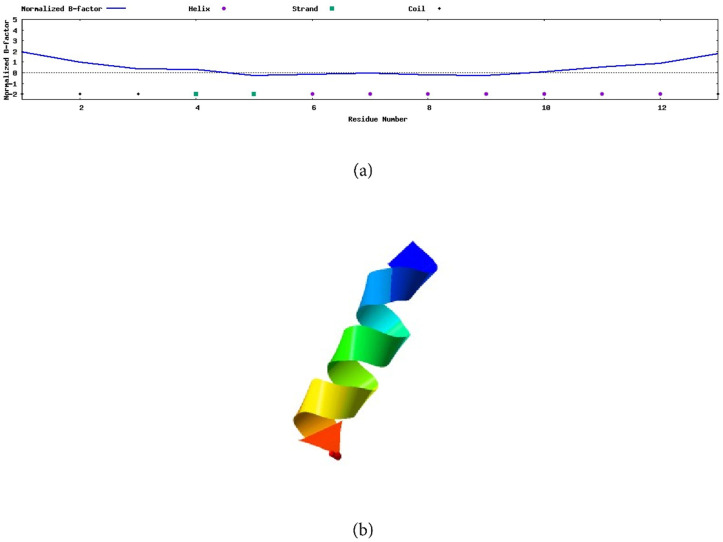
Secondary structure of the AXOTL-13 peptide from *Ambystoma mexicanum,* modeled by the I-TASSER server. (**a**) Prediction of amino acids involved in the formation of secondary structures. (**b**) Three-dimensional model of the secondary structure.

The prediction made by the I-TASSER web server for peptide AXOTL-13 indicates that the first three amino acids in the peptide sequence (GFSISLKRLQKML), glycine, phenylalanine, and serine, form a structure known as random coil, and the two successive residues, isoleucine and leucine, adopt a beta-sheet structure (Figure 4a). Similarly, the amino acids from position 6 to 12 configure an alpha helix, a secondary structure representative of the molecule. Finally, the amino acid in position 13 also forms a random coil. Besides that, the graph shows the estimated distance of the sequence to the secondary structure presented in each of the residues and the estimated model accuracy.

Consistent with the aforementioned, the 3D modeling created by this server, with a C-score of −0.24, clearly shows the formation of an alpha helix, and a random coil at one of the ends is noticeable (Figure 4b).

**Figure 5:**
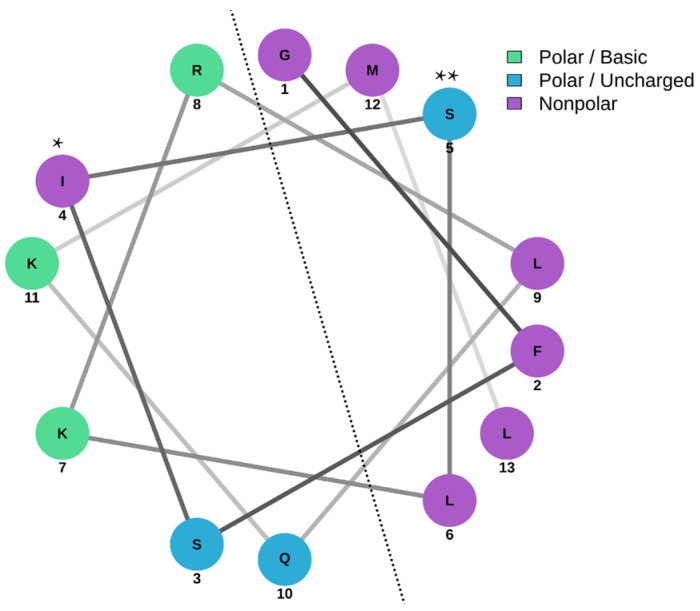
Schiffer–Edmundson wheel representation of the peptide AXOTL-13 from *Ambystoma mexicanum* simulated by NetWheels tool. Positively charged polar amino acids are shown in green, uncharged polar amino acids in blue, and nonpolar amino acids in purple. The dashed line differentiates the two faces of different polarity; *: isoleucine; **: serine.

The Schiffer–Edmundson wheel generated by the NetWheels tool allowed us to predict the location of amino acids and the amphipathic nature of their structure. In Figure 5, it is evident that on the right side of the helix, hydrophobic residues, represented in purple, predominate, while on the left side, hydrophilic residues, depicted in green and blue, prevail. However, the ideal amphipathicity of the molecule is disrupted by the presence of isoleucine, which is hydrophobic, in the hydrophilic portion, and by serine, which is hydrophilic, on the hydrophobic side.

### 3.6. Circular Dichroism (CD)

**Figure 6:**
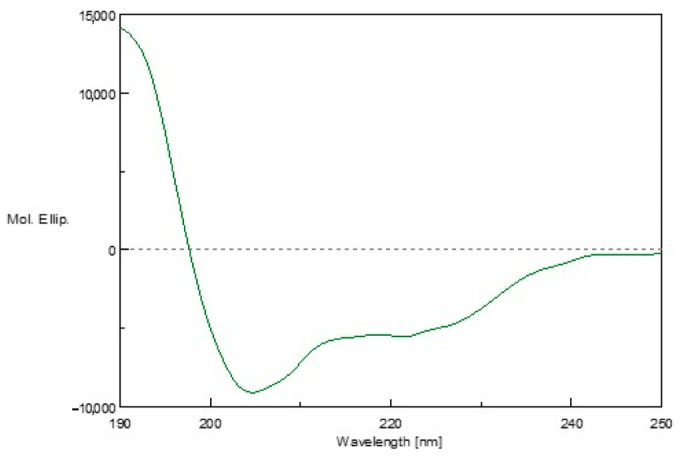
Circular dichroism analysis of the AXOTL-13 peptide from *Amystoma mexicanum*.

CD analysis was used to determine the secondary structure of AXOTL-13. In the 30% TFE solution, the spectrum exhibited a maximum before 200 nm and two minima between 200 nm and 230 nm, indicating an alpha-helical structure, consistent with the earlier prediction (Figure 6).

### 3.7. Scanning Electron Microscopy (SEM)

**Figure 7:**
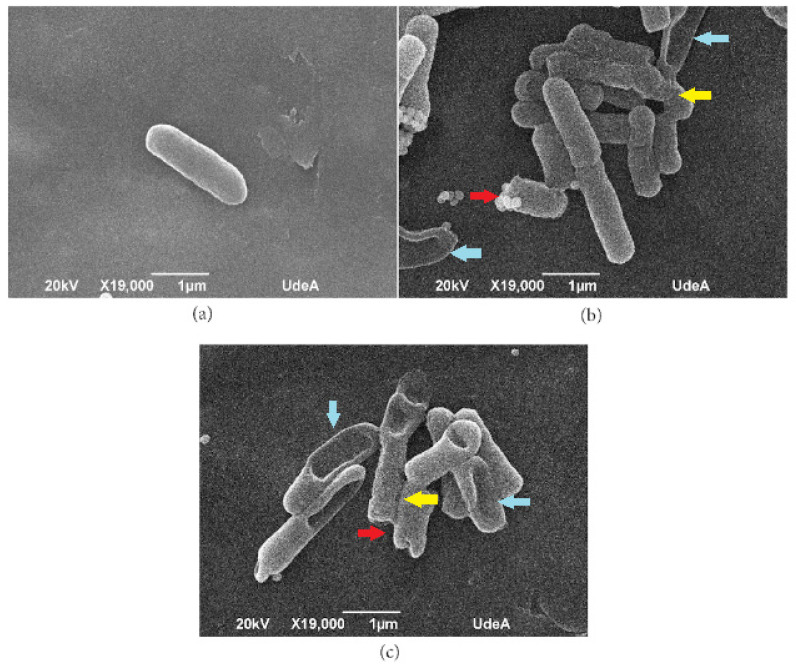
Scanning electron microscopy of the Gram-negative bacteria *Escherichia coli.* (**a**) C− (N/T): bacteria without treatment. (**b**) C+ (Ramosin): bacteria treated with 30 μM Ramosin for 15 min. (**c**) Bacteria were treated with 30 μM AXOTL-13 for 15 min. The yellow arrows indicate rough cells, the red arrows point to areas of shortening, and the blue arrows show indentations.

The SEM assay was conducted to observe the potential interaction between the AXOTL-13 peptide and the *E. coli* cell membrane, presumed to be its target of action. This assay allowed us to visualize the morphological changes caused by the peptide on the bacterial membrane. As seen in Figure 7a, cells in the negative control maintained their bacillary structure, appearing intact with a smooth surface. Cells exposed to the Ramosin peptide are observed to be damaged, rough, with indentations, and shortened, as shown in Figure 7b. Similarly, bacteria treated with AXOTL-13 exhibited the same characteristics (Figure 7c), suggesting that the peptide targets the *E. coli* cell membrane as proposed.

## 4. Discussion

The constant search for AMPs has resulted in the characterization of more than 3500 peptides in the APD3 database; however, only 4 of them have been identified in species of the Caudata order. In the *Andrias davidianus* salamander, two peptides with antibacterial activity have been identified: Andricin 01 and Andricin B ([47,48]. The Ramosin peptide, which also exhibits antimicrobial activity, has been identified in the *Bolitoglossa ramosi* salamander [31]. In *Ambystoma tigrinum*, an amphibian of the Ambystomatidae family, a composition of uncharacterized peptides with antibacterial activity was found [23]. However, to date, insufficient attention has been paid to the peptides in *Ambystoma mexicanum*, a species of the Ambystomatidae family. Although there are reports of peptides with potential antimicrobial activity, these have not yet been confirmed or evaluated, highlighting the need for more in-depth research in this area [24]. Therefore, this study represents the first report of a peptide in the *Ambystoma mexicanum* species with confirmed bactericidal activity, demonstrating the inhibition of *E. coli* growth by AXOTL-13 at a concentration of 35 μM and presenting hemolysis lower than 1.6% in erythrocytes at concentrations below 140 μM.

In the present research, the use of bioinformatics tools allowed the identification of potential peptides in the axolotl. These tools have already been implemented in previous studies [49,50], and their significance lies in the alternative they provide to costly and complex procedures that involve the direct use of animals for peptide extraction and purification [51]. The alignment revealed six peptide sequences potentially encoded by the axolotl, with possible antimicrobial activity, and expressed in its skin, intestine, and heart. The presence of these sequences in the skin could be attributed to the existence of granular glands in its dermis, which, according to previous studies, have proven to be the main secretors of active AMPs [52]. The presence of these peptides in the heart and intestine may be due to the epithelial cells and leukocytes found in these organs since both cell types are recognized for their ability to produce AMPs [18,53,54,55]. These findings demonstrate the diversity and importance of AMPs in the axolotl, suggesting that they play a crucial role in its immune response and its ability to combat pathogens.

The six peptides were subjected to an evaluation of their in vitro antimicrobial activity. However, peptides 4766, 4767, 4768, 4769, and 4771 did not exhibit the expected antibacterial effect. To understand the absence of the antibacterial effect, it is necessary to analyze the physicochemical properties essential for biological function, such as charge, hydrophobicity, amphipathicity, and the degree of secondary structure [56]. Additionally, it is necessary to investigate other possible biological functions besides antibacterial activity. AXOTL-13 was the only peptide to show antibacterial activity against one of the evaluated strains. It consists of a sequence of 13 amino acids (GFSISLKRLQKML) and has a cationic charge, possibly explained by the presence of basic amino acids such as lysine and arginine, and its GRAVY is 0.22, indicating the hydrophobic character of the molecule due to the six hydrophobic uncharged residues, including phenylalanine, isoleucine, leucine, and methionine, accounting for 46% of the amino acid sequence.

The secondary structure of the AXOTL-13 peptide corresponds partially to an alpha helix and a random coil, resulting from the amino acid sequence and its interaction with the environment. It is well known that certain amino acids, such as leucine, methionine, alanine, and glycine (which are present in AXOTL-13), favor the adoption of a helical structure [57,58]. Additionally, the structure displayed closely packed apolar side chains, creating a predominantly hydrophobic face opposite a hydrophilic face, thus indicating the amphipathic nature of the molecule [59]. This knowledge of the peptide structure enhances the understanding of how AXOTL-13 folds at the molecular level and serves as an essential tool to anticipate and predict, to some extent, its biological activity.

As mentioned earlier, the initial identification of the peptide AXOTL-13 was made possible through its alignment with Macropin 1 (MAC-1), showing 92.3% similarity. AXOTL-13 and MAC-1 share various physicochemical and structural properties, including length, net charge, and the adoption of an alpha-helical structure in a membrane-mimicking environment. Both AMPs exhibit an ideal amphipathic structure, altered by the presence of an amino acid with an opposite charge. Considering the above, it is expected that both peptides perform similar biological functions. Although it is not possible to directly compare the MIC of these peptides due to the experimental conditions (different bacterial growth media), both have antibacterial activity on *E. coli,* with an MIC of 35 μM in TSB for AXOTL-13 and 30 μM for MAC 1 measured in LB. Furthermore, AXOTL-13 shows a hemolysis percentage that does not exceed 1.6% at concentrations above 140 µM, while MAC-1 exhibits 50% lysis at 160 µM [60]. Of great relevance, the antimicrobial activity of other peptides derived from amphibians and salamanders shows similar and contrasting results concerning the hemolytic effect and MIC values required to generate an effect on bacterial strains related to those evaluated in this study. Thus, the peptide Andracin 01, isolated from the mucus of *A. davidianus* with an MIC of 8.3 µM, has antimicrobial activity against different strains of *E. coli* and, in contrast to our results, with an MIC of 33.5 µM, it did exert an effect on *S. aureus* [47]. On the other hand, the Andracin B peptide, derived from the bloodstream of *A. davidianus*, required an MIC of 16.7 µM to exert an antimicrobial effect on *E. coli* (CICC10293) [48]. Interestingly, the Ramosin peptide derived from the Salamandra *Bolitoglosa Ramosin* with an MIC between 17.5 µM and 35 µMn has a similar effect to AXOTL-13 against *E. coli* (ATCC 25922), also used in our study [31]. In contrast, the peptide Odorranain-NR, derived from the skin secretion of the amphibian *O. graham,* has an antimicrobial effect against *S. aureus* but no antimicrobial effect on *E. coli* (ATCC 25922) [61]. Similarly, all the peptides mentioned above as well as the AXOTL-13 peptide do not have an undesirable hemolytic effect. These results suggest that although the Axotl-13 peptide has an antimicrobial effect against *E. coli,* potential modifications in the template peptide sequence of this natural peptide may be required to improve its antimicrobial efficacy on the strains evaluated in this study and even on other bacterial strains of clinical interest not evaluated in the present study.

The antimicrobial and hemolytic activities of AMPs depend on multiple physicochemical parameters that are interrelated. Alteration of one parameter will cause changes in others, making it challenging to deduce the impact of a single factor on activity [62]. Despite this, understanding the functions and influences of these parameters provides insight into the peptide’s mechanism of action. One fundamental parameter in peptide activity is length, as the propensity to form secondary structures like alpha helices and beta sheets, which are essential for antimicrobial activity, decreases with decreasing sequence length. Therefore, the AXOTL-13 peptide, having a size considered favorable for activity, is easy to synthesize, making it a potential candidate for drug development.

On the other hand, it is well known that the interaction of AMPs with the cell membrane largely depends on electrostatic force, so the cationic charge of the AXOTL-13 peptide is key to explaining its selective attraction to bacterial membranes, which are negatively charged due to the presence of phospholipids, lipopolysaccharide, or teichoic acids. This is in contrast to erythrocytes, whose membranes contain higher proportions of zwitterionic phospholipids and cholesterol, partially explaining the absence of hemolytic activity. However, it is important to note that the hydrophobicity and amphipathicity of a peptide exert a more significant effect on the absence of erythrocyte attack than the cationic charge [62,63]. On the other hand, it has been demonstrated that an increase in cationicity enhances antimicrobial activity, while a parallel decrease in hydrophobicity leads to a reduction in hemolytic activity [62]. In the case of AXOTL-13, it can be inferred that its antimicrobial capacity is favored by its cationic charge, while its low hemolytic activity is related to its hydrophilic profile.

The observation of morphological changes through SEM confirmed the ability of AXOTL-13 to alter the structure of the *E. coli* cell membrane. This finding aligns with the traditional mechanisms associated with alpha-helical AMPs [63]. It has been documented that following the initial interaction with the cell wall, peptides that adopt an amphipathic alpha-helical structure are an ideal class of AMPs to interact with equally amphipathic biomembranes. This phenomenon occurs because the hydrophobic face interacts with the hydrophobic environment of the membrane phospholipids, leading to disorganization. However, our SEM analysis is not sufficient to elucidate the mechanism of action by which AXOTL-13 affects the bacterial membrane. Therefore, further functional studies should be carried out.

Thanks to the transcriptome analysis conducted using various bioinformatics tools and antimicrobial and hemolysis assays, the AXOTL-13 peptide was identified as a potential AMP. This cationic alpha-helical amphipathic peptide, as predicted, demonstrated the ability to inhibit the growth of *E. coli* (ATCC 25922) without inducing erythrocyte lysis. Further research is necessary to confirm the predicted physicochemical characteristics and elucidate the mechanism of action of this peptide. Additionally, exploration of other potential antimicrobial, antitumor, or immunomodulatory properties and the demonstration of in vivo antibacterial activity are warranted. Therefore, this study serves as a reference for future research in the field in the form of studies and working hypotheses. The findings and their implications should be discussed in the broadest context possible. Future research directions may also be highlighted.

## Figures and Tables

**Table 1:** Eligibility criteria for the selection of candidate peptides.

Parameter	Inclusion Criteria	Justification
Identity	Hits with a percentage lower than 100%	A 100% identity affirms that it is the same peptide as in the database.
Similarity	Hits with a percentage higher than 80%	The higher the similarity between two sequences, the greater the likelihood that they have a shared biological function.
Length	5–20 amino acids	Synthesizing short peptides can affect their stability due to the fewer peptide bonds. With a higher number of amino acids, more complications arise since a higher number of coupling steps is required.
Activity	Antimicrobial	Alignment with a peptide sequence whose antimicrobial activity has been previously tested.

**Table 2:** Characteristics of chemically synthesized peptides.

Peptide	Sequence	Molecular Weight (g/mol)	Net Charge	(%) Hydrophobicity	Similar Peptide from APD3	(%) Similarity with APD3 Peptide
4766	LIGGQLGGLIKAL	1252.8	1	53	AP02274	92.3
4767	YITGLIAPILKSL	1401.9	1	53	AP02275	92.3
4768	VLGSILGALKAI	1124.6	1	66	AP00521	91.7
4769	LHPLIGRVIGGVI	1343.9	1.25	53	AP00094	92.3
4770	GFSISLKRLQKML	1521.1	3	46	AP01956	92.3
4771	LLDTIGKIFGSLL	1389.9	0	53	AP00875	100

**Table 3:** Physicochemical properties of AXOTL-13.

Features	Description
Length	13 amino acid residues
Chemical formula	C_69_H_121_N_19_O_17_S
Extinction coefficient	0 $M^{-1}cm^{-1}$
Isoelectric point	11.65
Net charge	+3 *
Grand average of hydropathicity (GRAVY)	0.22

* At pH 7.5.

## Data Availability

Data is contained within the article and Supplementary Materials.

## Supplementary Materials

The following supporting information can be downloaded at https://www.mdpi.com/article/10.3390/pharmaceutics16111445/s1: Figure S1: Amplification of DNA sequences encoding candidate peptides from *Ambystoma mexicanum*, confirming their expression in skin, intestine, and heart. 1% agarose gel with PCR products stained with ethidium bromide corresponds to: (a) Peptide 4766, (b) Peptide 4767, (c) Peptide 4768, (d) Peptide 4769, (e) Peptide 4770, (f) Peptide 4771; Figure S2: Purity of peptides determined by reverse-phase liquid chromatography. (a) Peptide 4766, (b) Peptide 4767, (c) Peptide 4768, (d) Peptide 4769, (e) Peptide 4770, (f) Peptide 4771; Figure S3: Molecular weight of peptides determined by ionization mass spectrometry. (a) Peptide 4766, (b) Peptide 4767, (c) Peptide 4768, (d) Peptide 4769, (e) Peptide 4770, (f) Peptide 4771; Figure S4: Bactericidal effect of Peptide 4770 on *E. coli* (a) Triplicate of concentration 70 μM (b) Triplicate of concentration 35 μM.
