# Supplementary material for: Antibacterial Activity of AXOTL-13, a Novel Peptide Identified from the Transcriptome of the Salamander Ambystoma mexicanum
Source: Pharmaceutics. 2024 Nov 12;16(11):1445. doi: 10.3390/pharmaceutics16111445 (PMC11597150; doi:10.3390/pharmaceutics16111445)
Supplement: Supplementary file 1 [file pharmaceutics-16-01445-s001.zip › pharmaceutics-3263466-supplementary.pdf]

Supplementary information

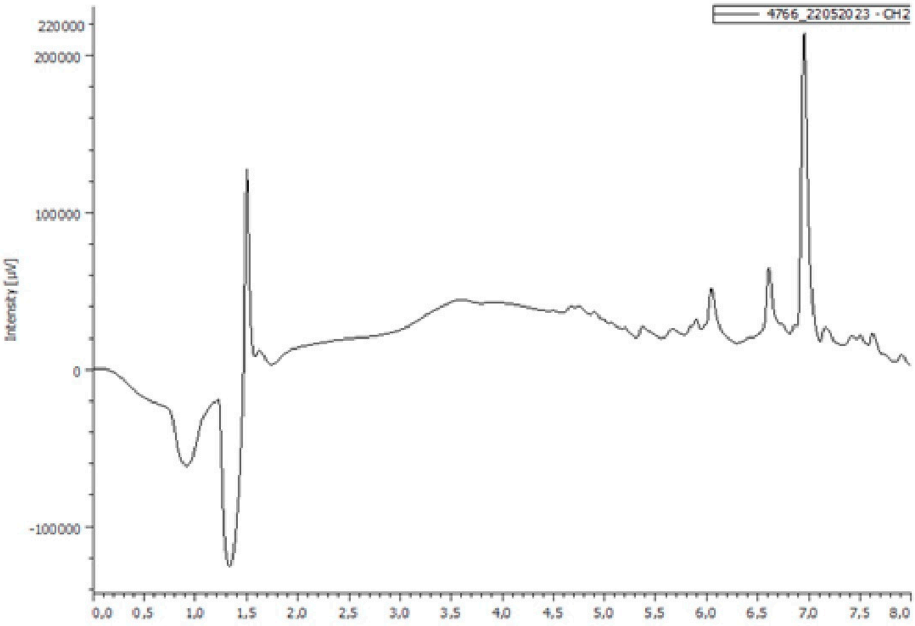

a

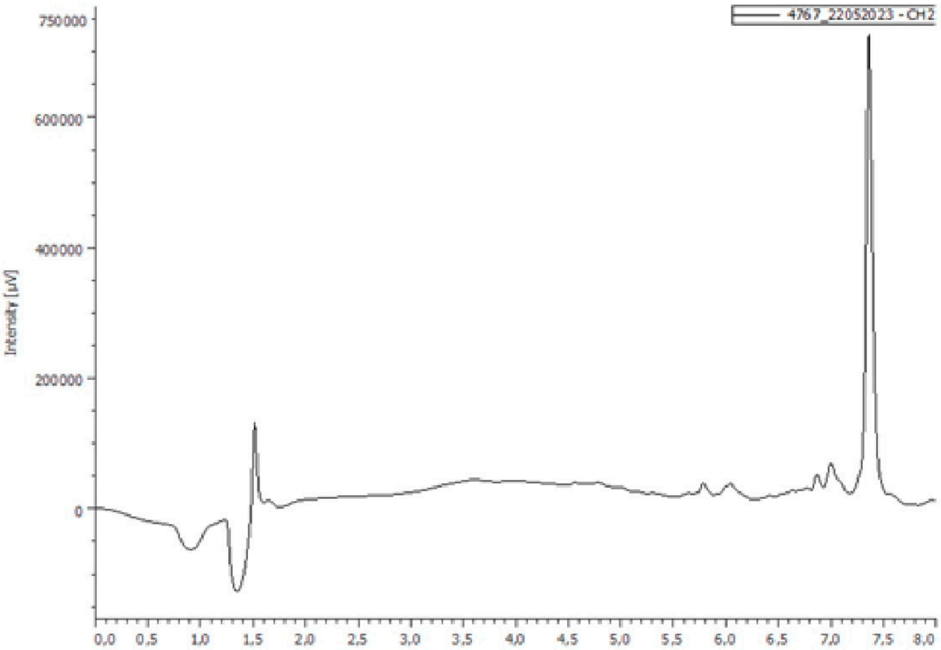

b

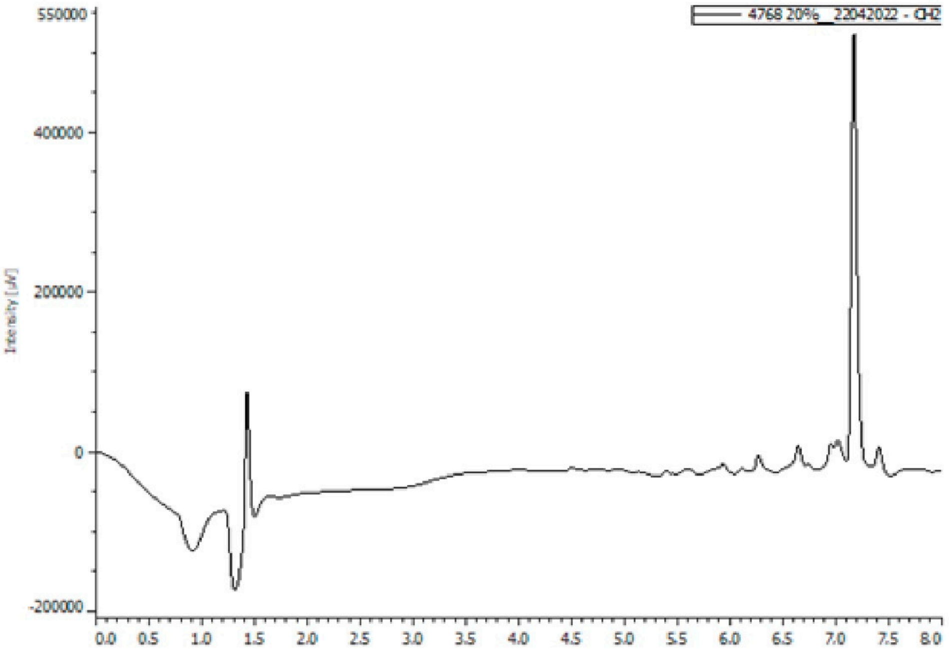

4768 20%__22042022 - CH2
550000
400000
200000
0
-200000
Intensity [µV]
0.0 0.5 1.0 1.5 2.0 2.5 3.0 3.5 4.0 4.5 5.0 5.5 6.0 6.5 7.0 7.5 8.0

c

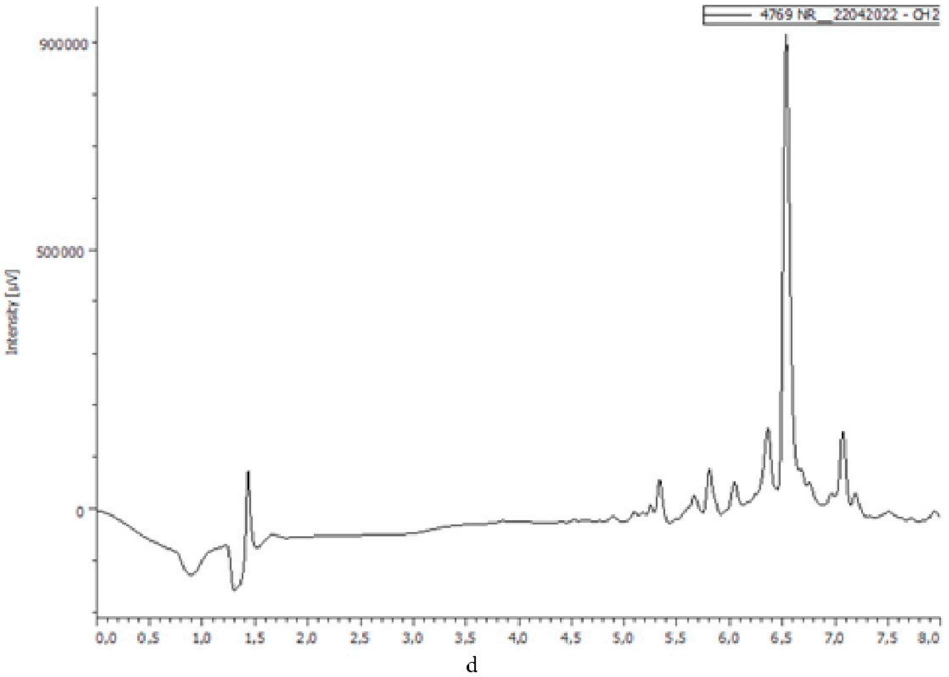

4769 NR__22042022 - CH2
900000
500000
0
Intensity [µV]
0,0 0,5 1,0 1,5 2,0 2,5 3,0 3,5 4,0 4,5 5,0 5,5 6,0 6,5 7,0 7,5 8,0

d

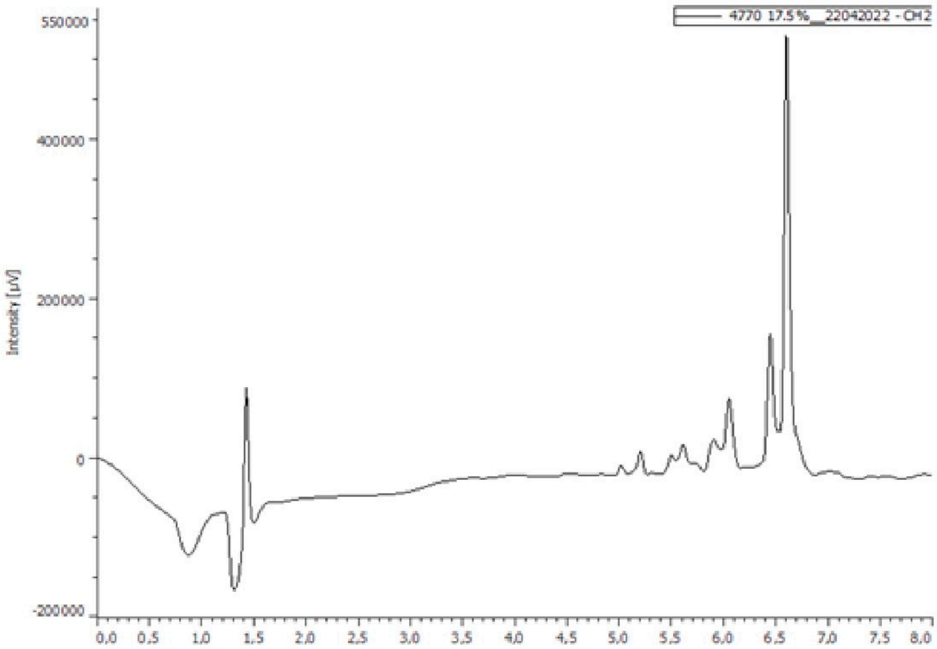

e

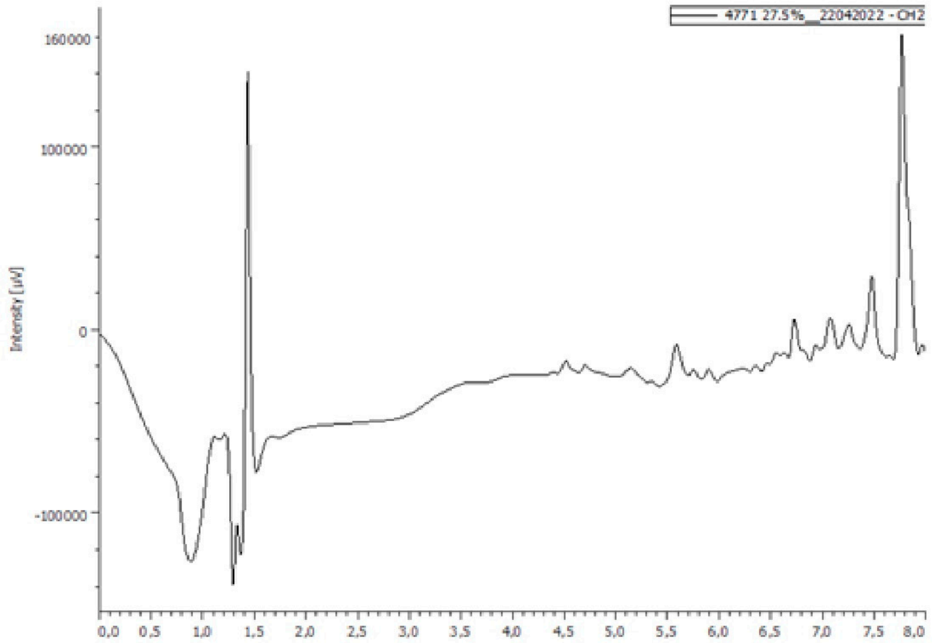

f

Figure S1. Purity of peptides determined by reverse-phase liquid chromatography. (a) Peptide 4766, (b) Peptide 4767, (c) Peptide 4768, (d) Peptide 4769, (e) Peptide 4770, (f) Peptide 4771**.**

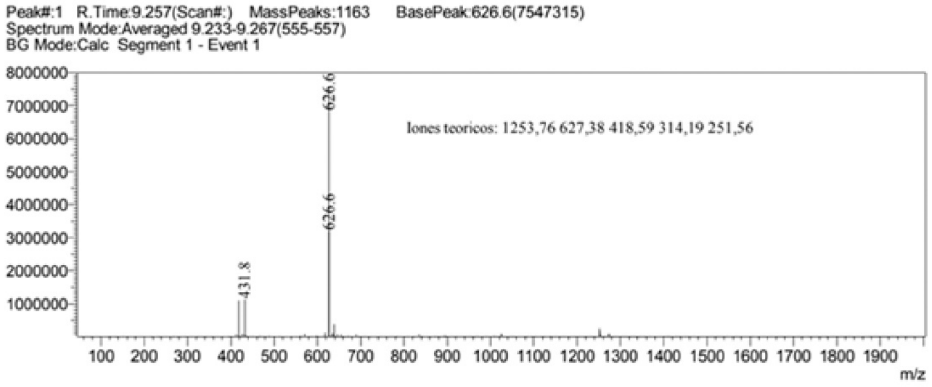
Peak#:1 R.Time:9.257(Scan#:) MassPeaks:1163 BasePeak:626.6(7547315)
Spectrum Mode:Averaged 9.233-9.267(555-557)
BG Mode:Calc Segment 1 - Event 1
Iones teoricos: 1253,76 627,38 418,59 314,19 251,56
626.6
626.6
431.8
m/z

a

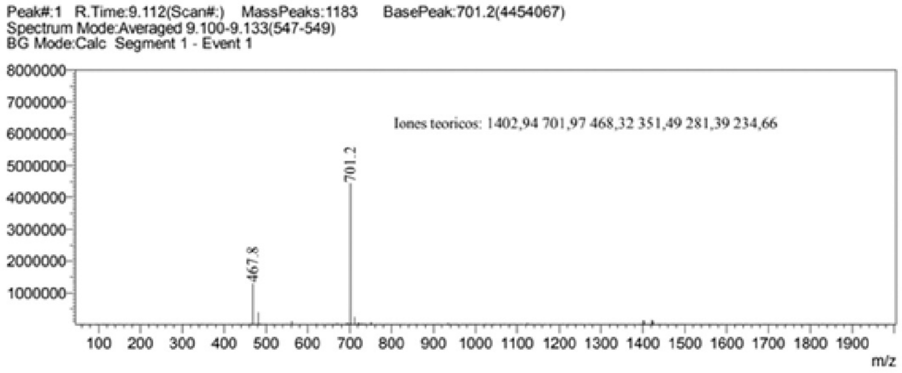
Peak#:1 R.Time:9.112(Scan#:) MassPeaks:1183 BasePeak:701.2(4454067)
Spectrum Mode:Averaged 9.100-9.133(547-549)
BG Mode:Calc Segment 1 - Event 1
Iones teoricos: 1402,94 701,97 468,32 351,49 281,39 234,66
701.2
467.8
m/z

b

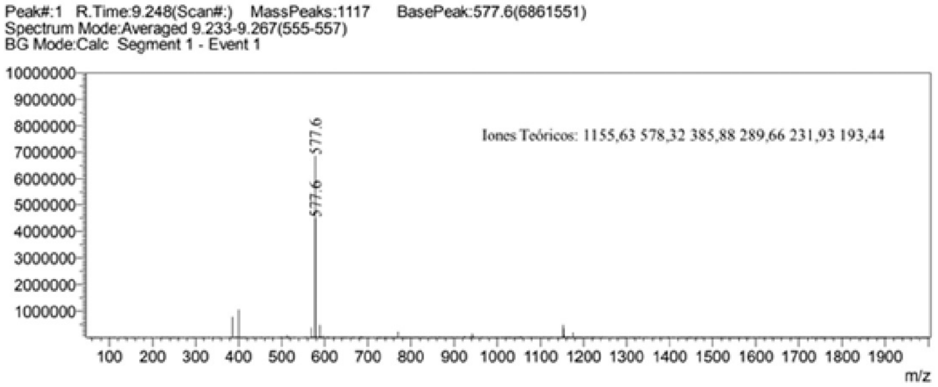
Peak#:1 R.Time:9.248(Scan#:) MassPeaks:1117 BasePeak:577.6(6861551)
Spectrum Mode:Averaged 9.233-9.267(555-557)
BG Mode:Calc Segment 1 - Event 1
Iones Teóricos: 1155,63 578,32 385,88 289,66 231,93 193,44
577.6
577.6
m/z

c

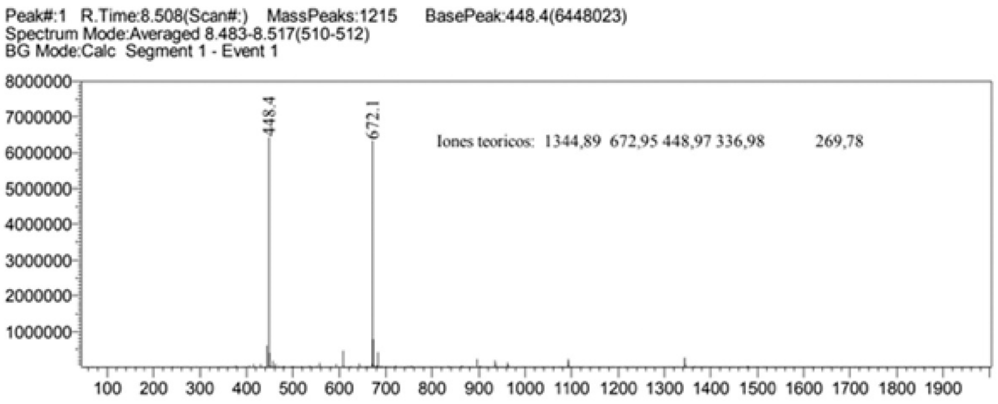

d

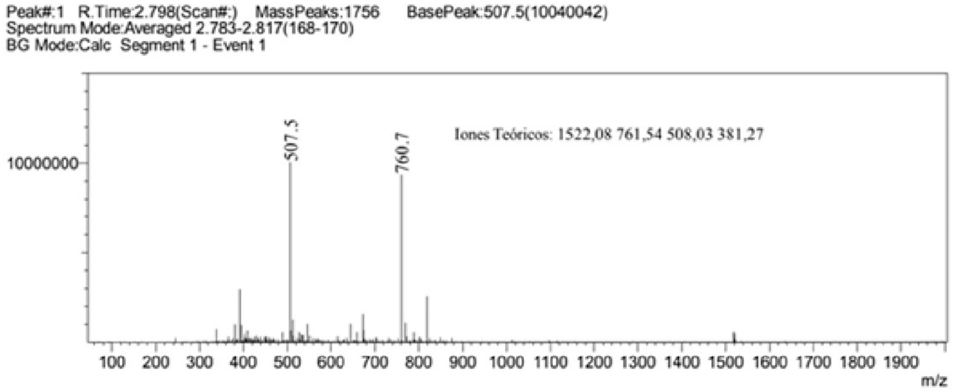

e

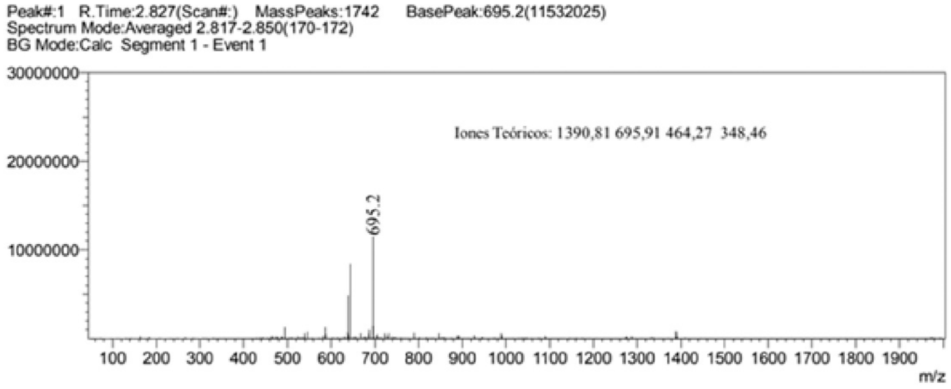

f

Figure S2. Molecular weight of peptides determined by ionization mass spectrometry. (a) Peptide 4766, (b) Peptide 4767, (c) Peptide 4768, (d) Peptide 4769, (e) Peptide 4770, (f) Peptide 4771.

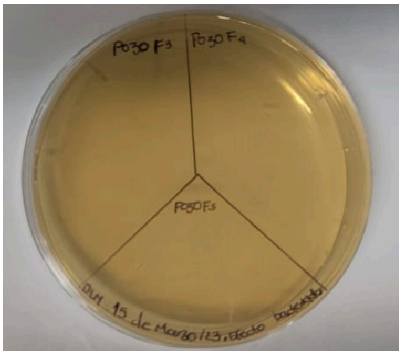

a

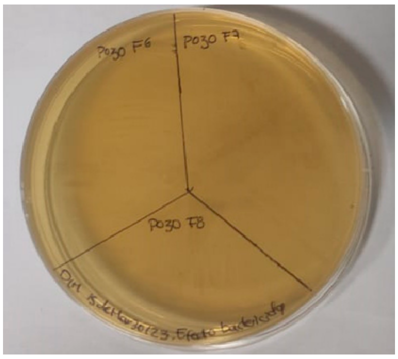

b

Figure S3. Bactericidal effect of Peptide 4770 on *Escherichia coli* (a) Triplicate of concentration 70 µM (b) Triplicate of concentration 35 µM.
